# Exploring a revised interprofessional learning curriculum in undergraduate health education programs at Linköping University

**DOI:** 10.1186/s12909-024-05458-3

**Published:** 2024-04-26

**Authors:** Elin A. Karlsson, Susanne Kvarnström, Maria Kvarnström

**Affiliations:** Department of Health, Medicine, and Caring Sciences, Linköping University, Linköping, Sweden

**Keywords:** Interprofessional learning, Interprofessional education, Curriculum, Curriculum development, Health professions Education (HPE), Interprofessional competence, Document analysis, Theory based evaluation

## Abstract

**Background:**

Interprofessional education aiming at providing competencies require evaluation in order to ensure that outcomes match the needs and ambitions. Health professionals today need a broad range of skills and competencies in order to provide high quality care, including interprofessional competence. Linköping University has been a pioneer in interprofessional learning for decades and this study provides one example of how a curriculum revision can be carried out. The aim of this study was to study the intentions and outcomes of a revised interprofessional learning curriculum in health professions education programs.

**Methods:**

This was a qualitative study, including documents (*n* = 143) and complementary interviews with key individuals (*n* = 4). Data included syllabuses, study guides, educational program plans, supervisor guides, and interview transcripts. A qualitative document analysis and a content analysis with a directed approach was used, applying a theoretical framework for curriculum development that guided the analysis.

**Results:**

The analysis resulted in one overarching theme named “A planned, lived, and attended curriculum” including four main categories inspired by a theoretical framework. The findings demonstrate a variety of aspects relating to the *why* and *how* of curriculum revision. The introduction of a programme director in interprofessional learning, with a mandate equal to respective program directors, seemed to contribute to legitimacy. Further, the partnership between the university and the healthcare sector had an impact on the curriculum revision, in that healthcare had a say in the revision regarding what suggestions to implement or not. The expectations of the teachers involved were high, although clear support structures seemed to be lacking.

**Conclusions:**

This study has identified some of the important links between teachers, organizational prerequisites, and healthcare when revising an existing fully integrated curriculum in interprofessional learning for health professions education programs. The aim of this curriculum revision was to legitimize and provide education that is up to date with current healthcare needs and to provide students with competencies to collaborate in teams to ensure patient safety. When redesigning a curriculum there seems to be a fine balance between pedagogical innovation and pragmatism. This study identified that the links provided between organizational support structures and the expectations on teachers were not aligned.

**Supplementary Information:**

The online version contains supplementary material available at 10.1186/s12909-024-05458-3.

## Background

Today’s Health Professions Education (HPE) needs to contain high quality learning activities that provide students from various programs the opportunity to learn with, from, and about each other in order for them to develop interprofessional competence [1]. This is a matter that is increasingly highlighted globally, in terms of what responsibility educating universities should have for facilitating students’ development of skills in collaboration and patient safety, where various professionals’ competencies might be needed [1, 2]. In parallel, interprofessional science has developed and established itself as an international research field over the last three decades [3]. Many HPEs around the world that support interprofessional learning (IPL) at their faculties use different learning approaches, sometimes including a fully integrated curriculum specifically for IPL. Without a fully integrated interprofessional curriculum as the umbrella, there are other approaches to organize IPL, often referred to as extra-curricular or partially integrated. Those include, for example, shorter courses, clinical rotations, and simulations with various numbers of educational programs participating. There are abundant examples of curriculum for IPL in the literature [4], including ways to develop IPL curriculum [5] and models for IPL [4]. However, previous research on IPL commonly focuses on students learning outcomes and factors that may facilitate, or hinder, implementation [6, 7] rather than *how* a curriculum is created, implemented, performed, and revised. There is a need for studies on *how* curriculum development is performed, and there is a lack of studies exploring the changes in a curriculum over time. In Sweden there are national degree objectives regarding teamwork and collaborative competences for all HPEs. Thus, the development of curriculums in IPL is important in order to ensure that HPE students enter the labor market with appropriate interprofessional skills. This is essential for their colleagues, employers (i.e., healthcare organizations) and patients.

### The IPL-curriculum of Linköping University

At Linköping University, the medical faculty has been a pioneer regarding IPL, which has been a clear feature since its origin, in 1986 [8, 9]. There is a shared model for IPL at the faculty: a fully integrated curriculum for IPL. The curriculum includes three modules that are designed to promote a progression of IPL throughout the HPE programs, mandatory for all participating students. Further, at Linköping University, problem-based learning (PBL) and student-centered learning are central features whereby students commonly work in groups with so called scenarios (i.e., cases meant to spur questions, discussions, and learning) [10]. In 2012, the medical faculty addressed the need for reforms in how IPL was organized and facilitated and an investigation to produce solid suggestions for how to perform such a curriculum development was initiated [11]. One of the major reasons for this project were to relate to global changes to a new generation of teachers and students, as well as an increasing number of students, to address healthcare needs which called for strengthened interprofessional education [12]. This work resulted in a report [12] that was the foundation for the curriculum development. To establish how to modify and carry out educational activities in IPL, a committee with representatives from all of the involved programs was appointed to this work. These programs were within biomedical laboratory science (BMLS), medical biology, medicine (M), nursing (N), occupational therapy (OT), physiotherapy (PT) and speech and language pathology (SLP). The last iteration of the bachelor’s program in medical biology began in the autumn semester of 2017 and was replaced from autumn 2018 by another bachelor program within biomedicine. The new biomedicine program, which is international and thus uses the English language, does not participate in the current IPL curriculum. Thus, the current IPL curriculum at Linköping University includes six different HPE programs at the medical faculty. When using the term “medical faculty” we, thus, refer to the faculty at the University in which all the above mentioned HPE programs are situated. Parallel with the implementation of the revised IPL curriculum, the medical program was decentralized to multiple sites, requiring additional organizational structure. This is enacted before the second IPL module, and these medical students, may thus, perform the module in either of three different healthcare regions (previously called county councils) in Sweden (and four different cities), as in the third module.

Since the implementation of the current IPL curriculum in autumn 2016, the curriculum encompasses 8 weeks of full-time studies in total. The three modules are Professionalism in Healthcare (6 credits), Quality Improvement and Learning in Practice (3 credits), and Professional Perspectives in Collaboration (3 credits). The first module, referred to as IPL1, focuses on professionalism in healthcare and the common denominators for future healthcare workers, such as having a holistic biopsychosocial perspective on health and the common values based on regulations and ethical principles. The majority of the HPE programs attend this module during their first semester (see Table 1). The second module, referred to as IPL2, has a slightly different focus as the students are supposed to learn about improvement science and are assigned a quality improvement scenario in a practical healthcare setting. Commonly this is carried out during the end of the students’ education. The third module, referred to as IPL3, focuses on professional perspectives in collaboration at interprofessional training wards (IPTW) and interprofessional training primary healthcare centres where students are stationary at the ward/center throughout the placement and work in teams to plan and deliver the care of the patients. The wards and centers are driven by the healthcare regions with clinically active team supervisors, employed by the regions, and most but not all programs participate (see Table 1). The third module run in 2-weeks periods consecutively over the semester. At the IPTWs and student are responsible for the full care of the patients at the ward, under supervision by profession specific supervisors as well as team supervisors (more details about the IPTWs with the ones in Linköping as an example are described by Törnqvist et al. [13]. Commonly, the HPE programs participate in the modules in numeric order, with exception of the medical program that has the third module before the second.

**Table 1:** Overview of the IPL modules, stated in current semester (sem.)

Program	BMLS	M	*N*	OT	PT	SLP
Module	Sem.	Sem.	Sem.	Sem.	Sem.	Sem.
**IPL1** Four weeks	1	1	1	1	1	2
**IPL2** Two weeks	5	11	5	5	5	5
**IPL3** Two weeks	6	9	6	6	6	-

The programmes included are biomedical laboratory science (BMLS), medicine (M), nursing (N), occupational therapy (OT), physiotherapy (PT) and speech and language pathology (SLP)

Alongside the activities in the three different IPL modules, there are also other interprofessional learning activities involving students from two or more different programs, such as interprofessional simulations and clinical activities.

This study provides an example of how a fully integrated curriculum in IPL can be revised and staged over time. The study also uses a theoretical framework for curriculum development [14], appropriate for theorizing the findings and for facilitating that others may benefit from the lessons learnt during the process at Linköping University.

#### AIM

The aim of this study was to study the intentions and outcomes of a revised interprofessional learning curriculum in HPE programs.

### Research questions

1. Why and how was the current curriculum revised?
2. How can the intentions and outcomes of the curriculum revision be understood and theorized in a model for curriculum development?
3. What lessons are to be learnt from this work and what areas need deepened knowledge?

## Methods

### Study procedures

#### Theoretical framework

This is a qualitative document study together with supplementary interviews with key individuals, in which we used a theory-based evaluation in accordance with Lilliedahl et al. [15]. Further, to facilitate the interpretation and theorizing of empirical findings, we used a theoretical framework derived from the four interrelated dimensions for curriculum development processes as described by Lee et al. [14], i.e., (1) Identifying future orientation of Health practices; (2) Defining and understanding capabilities; (3) Teaching, learning and assessment; and (4) Supporting institutional delivery [14]. These dimensions were used as a structured tool to facilitate the interpretation and theorizing of empirical findings.

#### Material

“Material culture”, such as documents, records, artefacts and archives, provide a valuable source of information regarding organizations and HPE, since they can give a behind-the-scenes look at different processes [16]. In this study, we used documents and texts from different periods of time as well as different sources.

As supplementary data, to further expand the understanding of some of the elements that emanated during the analysis of the documents, four individual interviews were performed with key persons who were active in the curriculum revision in 2014–2016.

### Context of the documents

When using documents as data, it is important to define their context as well as their original purpose and who documented them [17, 18]. The documents used in this study served primarily as the foundation for students’ education in terms of educational program plans, syllabuses, and study guides. These kinds of documents have been documented primarily for the students undertaking the HPE programs but are also eminently relevant for the teachers involved. These documents are generally written by teachers, and management of the specific HPE programs whereas program and course syllabuses are determined by the educational board of the medical faculty. These two kinds of documents are more formal in their character and include the learning objectives for each specific course during the whole educational program. There is one overarching program syllabus for each program, and these have been included in the analysis. The course syllabuses were collected for each program and each course in which one of the three IPL modules was integrated. A study guide is a document that is directed solely from the course management within each program, or within each IPL module, and is thus more flexible in terms of revisions. Apart from the goals of the specific course, the study guide also includes more detailed content regarding the current learning activities, examination forms, and the organization of these. There are study guides for both the program-specific courses and for the unique IPL module that is organized within the programs’ specific courses. These study guides were collected for the specific IPL modules, as well as for the program-specific courses in which the IPL modules are integrated. For the third module of IPL, information is generally derived from both the university’s and the region’s digital platform, in which the specific care units and care centres share information directed towards the students placed there. Thus, this information tends to differ as it is context bound.

We have also included teacher guides for the IPL modules, which is a document written by the course management, for the teachers and tutors who are assigned to work in one or more of the learning activities involved. Further, in order to be able to capture the intentions and arguments for the revision of the curriculum, we chose to include formal decision documents and directives for the implementation of the present curriculum, written by dean and the faculty board of the medical faculty.

#### Timeline of the documents

The documents were collected in accordance with a specific timeline, as illustrated in Table 2 below: Phase (1) Formal decisions and directives from the planning process during the years 2012–2015; Phase (2) Before the curriculum revision, during spring semester in 2016; Phase (3) The semester in which the revised curriculum was first implemented, autumn 2026 to autumn 2021; and (4) The current design of the modules, spring semester 2022, as some parts may have been tried out and further revised. An overview of the documents is presented in Table 2. In total, 143 documents were included, representing four different time phases that will be referred to in this paper in order to facilitate keeping track on the time aspect and the context of these documents. The number of documents differs throughout the phases. This is due to, for instance, the fact that some of the older documents were not found in the archive.

**Table 2:** Overview of the included documents in the analysis, from four different time phases

Phase 1 Decisional documents and planning from the years 2012–2015 *N* = 8	Phase 2 Documents from the last semester of the old curriculum, spring 2016 *N* = 47	Phase 3 Documents from the first semester of the revised curriculum, on each program and module, autumn 2016 – autumn 2021 *N* = 32	Phase 4 Current documents, from spring 2022 *N* = 56
Formal decisions, directives and reports with suggestions and decisions.	Documents *before* the implementation of current curriculum. Syllabuses and study guides for the respective HPE program and the three IPL modules, program syllabuses from each HPE program. Study guides and teacher guides for each module.	Documents from the study guides and teacher guides for each IPL module when the current curriculum was implemented for the first time. Also, course syllabuses and study guides for the respective HPE programs for the courses in which the IPL modules are integrated. Naturally the time span for collection of documents from the different HPE programs varies. For instance, the first IPL module was implemented in its current form during autumn 2016, for all HPE programs except for the SLP program where students attend spring semesters only, i.e., participated in the current curriculum spring 2017 for the first time. Further, the revised modules taking place later in the educational program were not launched until some semesters after the revision.	Study guides and teacher guides for each IPL module. Course syllabuses and study guides for the respective HPE program and the three IPL modules, and educational program plans from each participating HPE program.

### Interview participants

Supplementary data were collected using a purposive sampling strategy [19], to obtain perceptions from people who held positions with a mandate at a significant executive level, and who were involved in the developmental process of the revised IPL curriculum. Four key individuals were invited to participate in separate interviews. Written information about the study was given, and all four agreed to participate. All participants had clinical backgrounds as healthcare professionals. All interviews were performed by SK and conducted during May 2023. The interviews were carried out either in the participants’ work premises or in their home and lasted between 60 and 90 min. The semi-structured interview form consisted of, apart from background data, each respondent’s perceptions of the process, and specific questions that had arisen in connection with the initial document analysis, such as when and why the respondent perceived that a certain learning activity had been replaced in syllabuses (Appendix 1).

### Data analysis

Data were analyzed using a qualitative document analysis [18] together with a content analysis using a directed approach [20] where the four dimensions of Lee et al. [14] constituted the theoretical framework. Our analysis included features of both manifest and latent data, and a process including skimming, careful reading and interpretations of patterns within the data. The process requires focused re-reading and review of the coding and category construction, to uncover themes relevant to a specific phenomenon [18]. In this study, documents were initially skimmed, then read more carefully as meaning units were discovered and categorized. The first author performed these steps, in the software NVIVO [20], version 14. Four main categories were created in accordance with the chosen theoretical framework (i.e., Lee et al., [14]), relating to (1) The future orientation of health practices; (2) The desired competencies and capabilities for the students; (3) Activities for teaching, learning and assessment; and (4) Organizational requirements, supporting institutional delivery [14]. Initially, all authors read and coded a selected set of documents and discussed the coding and categorization of these in order to calibrate and to ensure a purposeful analysis. The analysis was thereafter performed by the first author and discussed with the other authors. The preliminary analysis file in NVIVO was shared with the second author and another researcher who went through a selection of the meaning units to review and refine the categories and to ensure that no meaning units of relevance were omitted. Within the four main categories based on the dimensions, subcategories were then developed. The process moved from being deductive to inductive, back and forth as categories were revised and new subcategories identified. The analytical process was continuously discussed with all authors during physical meetings in which the first author showed the NVIVO file on a large screen, including the coding of meaning units, ideas for subcategories, and noted suggestions for quotes. In this study, the usage of NVIVO facilitated a transparent process in which all authors to some extents were involved in the analysis. The analysis of the documents generated unresolved questions that the collected documents could not answer. These questions were noted and added to the interview guide. Further, based on the questions that had emerged during the document analysis, we decided on who we needed to invite to gain a deeper understanding of these specific questions about the curriculum revision. The labels of each main category and subcategory were revised to mirror the empirical material in it, and representative quotes were selected. The four main categories yielded were (1) Curriculums in interprofessional education within healthcare professions – an extended matter of providing high quality care to patients; (2) Interprofessional competences and goals expressed increasingly coherently over time; (3) The design of learning activities in an interprofessional curriculum; and (4) Organizational prerequisites when staging the current curriculum – a transformed distribution of responsibilities and structure.

The supplementary interviews were recorded and transcribed verbatim, generating 106 pages of data. The text of the interviews was analysed using content analysis with a directed approach [21], based on the theoretical framework of Lee et al. [14], and preliminary categories were developed. The preliminary categories were then merged into the categories identified in the document analysis described above. Because the documents were analysed first, and included an extensive amount of data, the interviews were a complementary strategy primarily intended to fill some of the knowledge gaps. However, these interviews yielded interesting findings and all the data that was relevant for this study in accordance with the four dimensions of Lee et al. [14], was, thus, included.

### Ethical considerations

Verbal consent from interview participants was obtained, including assurances of confidentiality and of participant withdrawal from the study at any time without any explanation whatsoever. To further protect the confidentiality of the participants, transcribed interview excerpts do not appear in the Result section. The collected data are securely stored in password-secured computers and not shared beyond the research group. In accordance with the advisory remark from the Swedish Ethical Review Authority, ethical approval for this study was not needed (Dnr 2022-06875-01).

## Results

The analysis resulted in one overarching theme named “A planned, lived, and attended curriculum”, including four main categories inspired by Lee et al. [14]. The first: “Curriculums in interprofessional education within healthcare professions – an extended matter of providing high quality care to patients”, included the arguments for developing and nurturing IPL for healthcare professionals, commonly referred to as being a crucial matter for ensuring that patients are given optimal and appropriate care. The second category, “Interprofessional competences and goals expressed increasingly coherently over time”, related to how learning outcomes and desired competencies in IPL have been articulated increasingly coherently between programs over the years. The third category, “The design of learning activities in an interprofessional curriculum”, reflected how learning activities were designed and revised before and during the current curriculum. Lastly, the fourth category, “Organizational prerequisites when staging the current curriculum – a transformed distribution of responsibilities and structure”, described the organizational challenges and structures in the context of the IPL curriculum, in which the role of teachers was prominent.

### Curriculums in interprofessional education within healthcare professions – an extended matter of providing high quality care to patients

This first category was derived primarily from decisional documents and directives from the university at an early stage (phase 1, see Table 2). However, the motives for, and benefits of, IPL were also highlighted in course documents in phases 2–4, on each HPE and module. One aspect that brought about the curriculum revision was a striving for unity in study guides and syllabuses, and the intention that the IPL modules should be looked upon as an integrated part of the respective program, i.e., not something that stood out or were perceived as peripheral to the program-specific content. In particular, it was desired that the interprofessional competence should be seen as part of the professional competence. Different professional cultures and norms were visible through the documents, before the curriculum revision (i.e., phase 2, see Table 2), as IPL and its content tended to be described in various terms and were permitted various amounts of space in the respective programs’ course documents. The aspect of uniformity as a driving force to revise the IPL curriculum was also visible in the interview data with key persons. The following quote illustrates one strategy to achieve unity across programs, in terms of a standardized text that was implemented within the curriculum revision in all of the six participating programs educational program and course syllabuses, for all the courses in which an IPL module was included:

‟Interprofessional learning means that students from several professions learn with, about and from each other. This form of work stimulates and supports the students’ development of professional competence and prepares them for interprofessional teamwork and collaboration in the future professional practice.” [The quote can be found in all of the six participating programs’ syllabus, for the courses where the three IPL modules are included, and in their educational program plan at spring semester 2022, i.e., phase 4].

In terms of an IPL curriculum specifically designed for future healthcare professionals, there were prominent arguments relating to the importance of IPL and teamwork with reference to the quality of care, patient safety and as something that is necessary in order to manage the demands of future healthcare. Early documents (from phase 1) emphasized that IPL in general was significant for global health and innovation. This was also reflected in some syllabuses and educational program plans in some programs, through phases 2–4, although to a limited extent. The presentation of IPL as significant for good care and patient safety seemed to be emphasized even more in the more recent documents of current curriculum and could be observed in the documents from the spring semester 2022 at several levels directed towards faculty management, teachers and students. These findings are closely related to research questions 1–2, in terms of why the curriculum was revised and how these intentions can be understood and theorized.

### Interprofessional competences and goals expressed increasingly coherently over time

The second category concluded that although learning objectives and assessment criteria only have changed marginally since the introduction of the current curriculum, there were differences in how the interprofessional goals and the program specific goals and competencies were written, and given space in the documents. As mentioned in the previous category, IPL was given various amounts of space in the respective programs’ course documents. This was also reflected upon goals and expected learning outcomes, and how and where these are articulated. For instance, during phase 2, the goals and content of IPL was sometimes highlighted first in a syllabus, even though IPL consists of fewer credits compared to the program specific parts of the course. On the contrary, some programs chose to refer to the interprofessional content as “other”, with reference to an appendix at the end of syllabus. Before the curriculum revision (phase 2), this was quite prominent and in line with previous examples. Nowadays (phase 4), differences of the same magnitude do not occur, although they have not completely gone. Instead, the learning objectives in the programs’ syllabus were outlined together, without distinguishing between what concerns an interprofessional module and what does not and are now structured in accordance with the three domains ‛knowledge and understanding’, ‛skills and abilities’, and ‛values and attitudes’, i.e., the European system for increasing coherence in higher education: Bologna. The findings of the second main category primarily answers research questions 1–2, by demonstrating the intentions and outcomes of interprofessional competencies and goals, and how the goals were revised in the curriculum. A lesson learned (relating to research question 3) is that syllabuses and other course documents can be valuable tools for reducing variations between programs and for increasing cohesion and clarity in an interprofessional curriculum.

Furthermore, the medical faculty’s profile regarding interprofessional education appeared in the programs’ educational program plans both before and after the curriculum revision, thus phase 2 and 3–4, where one of the local goals for education programs at the medical faculty at Linköping University was for the students to ‟have achieved interprofessional competence …”. However, this local goal has been adjusted between spring 2016 (phase 2) and spring 2022 (phase 4). Previously, the end of the goal read ‟"… to increase employability”, which were changed to ‟… to be able to work in teams with other professional groups”, which is increasingly in line with the intentions and arguments of the curriculum revision as described in the first category.

### The design of learning activities in an interprofessional curriculum

The third category describes how the problem-based approach, central at the medical faculty at Linköping University, was justified in reports and decision documents as supporting deep learning and an interactive learning environment.

‟Problem-based learning thus moves from example to theory, unlike traditional education, which often starts from a principle or theory that is then illustrated with examples … a better strategy for deepening the student’s understanding than a lecture-based educational design, where the relevance of the theoretical concepts can be more difficult to discern.” [Report, 2014, phase 1.]

The content included in the IPL modules is similar nowadays as before the curriculum development, although the forms of learning activities have been modified. Regarding the two latter IPL modules, the changes were minimal, except that the interprofessional training wards have been expanded with interprofessional training centres in primary healthcare. Learning activities commonly consisted of group work, seminars and lectures that concern topics relevant to future healthcare professionals, such as ethics, health theories, and improvement science (see Table 1). However, learning activities did not include theory about IPL, and what it is, but the students instead gathered around other topics that they were reading.

In referral letters from phase 1, commenting on the suggested curriculum revision, the programs raised the question early of how the interprofessional modules should successfully fit together with the program-specific content that is provided in parallel at the beginning of the students’ education and, to some extent, also regarding the second IPL module. There was a request for greater clarity in how content and scheduling should be integrated with the program-specific content. At the start of the revised curriculum, one learning activity that was stated to support the integration between IPL and the program-specific content was that the scenarios for the IPL group also reappeared in the program-specific groups, albeit with a different focus, referred to as “cut-outs”. However, in present-day syllabuses (i.e., phase 4), the scenarios for the IPL student groups were no longer repeated in the subsequent program-specific groups. One of the findings in the analysis of the supplementary interviews with key individuals was perceptions of difficulties for new teachers to understand the new integrated pedagogical ideas and to explain them to students, and for the IPL management to maintain a perceived complex learning activity design over time.

In addition, change have occurred also *within* the current curriculum, (phase 3 to 4). For instance, the number of seminars has been reduced. When the second IPL module was planned in phase 1, the suggestion was to change the content and let the student group meet simulated stroke patients and have simulation training in teams. Syllabuses from the current curriculum (phase 4) show, however, that this was not adopted, and that the focus on improvement science remains. Letters of comment from phase 1 demonstrated that the suggested removal of improvement science received resistance, in particular from representatives from regions and municipalities, indicating that they wanted to retain improvement science as they viewed this as a natural and important part of IPL, and an essential competence for healthcare professionals. The established cooperation between the university and healthcare was, according to the interviewed key individuals, a powerful factor that contributed to *not* changing this IPL module. Also, an initial idea with portfolio as a pedagogical tool and examination, seems to have become fragmented in phase 4. In early letters of comment (phase 1), both teachers and students highlighted a concern about how such a portfolio examination would be assessed, and whether this would be legally secure and fair. The findings from interviews with key individuals indicated that the portfolio was perceived by teachers as a complex task to assess, and in particular to use as an examination with the formalities connected to an exam. This caused insecurity and frustration among teachers and may, thus, have caused difficulty for the IPL management to, what one key individual illustratively referred to as “balance pedagogical innovation and pragmatism”.

Further, although it was rare, it was notable that some programs have managed to integrate goals and learning outcomes related to IPL in program-specific assignments (in phase 4). The occupational therapy program, during its fifth semester, has integrated learning outcomes from the second IPL module in program-specific content by acknowledging interprofessional collaboration between different stakeholders within vocational rehabilitation. The syllabuses could, in this way, reveal variety in how the programs have designed their courses that include an IPL module, and in their strategies to integrate the program-specific content with the interprofessional content, beyond what is already included in the IPL module. Lessons learned from this category (research question 3) relates for instance to the need for sensitivity and flexibility with regards to the teachers and stakeholders involved. This category puts the outcomes (research question 2) of the curriculum revision in a different light, reflecting the necessity of pragmatism.

### Organizational prerequisites when staging the current curriculum – a transformed distribution of responsibilities and structure

The fourth category contains the contextual conditions that affected the staging of the interprofessional modules. The following content reflects all three research questions, in terms of how the curriculum revision was carried out, the organizational outcomes of it, and what we can learn from this process. A clearly prominent feature was the demands on teachers’ competence, as well as the organizational challenges that the revised curriculum entailed. For example, the revised curriculum meant that the programs were given greater responsibility for the IPL modules. It was desired that the teachers who were involved in the first semester also would be involved in the first interprofessional module, in order to create a more cohesive feeling of IPL and the program-specific elements. Previously, on some programs, this had been arranged separately by a small group of teachers involved in IPL and another group of teachers in the program-specific elements. Further, PBL was previously introduced during the first interprofessional module and thus included in the credits related to IPL. However, due to the curriculum revision, PBL had to be introduced in the respective programs before IPL (starting in phase 3), which contributed considerably to an increased responsibility on the programs when it comes to introduction of the pedagogical framework PBL. In letters of comment (phase 1), there was a concern that the introduction of PBL would take valuable time away from program-specific content. It was also considered important that all students received the same introduction.

The documents also revealed that the curriculum revision led to the introduction of a programme director for IPL, at the same hierarchical level as the respective programs’ directors, as decided by the faculty deans. One of the findings in the analysis of the interview data was perceptions that this decision constituted a vital step that provided a clear signal from the faculty management to the programs that IPL was equally important. It also provided a forum for continuous discussion and meetings which have been beneficial for preserving the interprofessional perspective on various matters.

A total of six programs are now included in the interprofessional modules (see Table 1), and there have previously also been intentions to include other programs, such as the psychology program and the sociology program, which have not been realized. Before the introduction of the current curriculum (phase 2), the interprofessional training wards (i.e., the third IPL module) were located at hospitals run by the same region. But since then, the medical program has been decentralized to other locations after which interprofessional training centres have started in healthcare settings operated by the local regions also in these locations. This has led to organizational differences as students now carry out the third IPL module with a varied range of healthcare students who come from other universities.

#### The role of the teachers – expectations, requirements and support

The role of the teachers is characterized by demands and expectations placed on teachers through the curriculum revision, in all phases, and the support available to them for meeting this need. An early decision document concluded that it was important to develop how teachers are introduced to the IPL modules:

*‟How teachers are introduced to the modules of IPL and are constantly given the opportunity for a continuous education to participate in the development of these modules.””* [Decision document of Linköping University in 2012, phase 1.]

The changes that the current curriculum entailed contributed to an increased need for competence development in teachers and new forms of administration. The document analysis revealed that a course for teachers was created in 2015. However, data obtained from interviews with key individuals indicated that the request for teachers to participate in such a course was perceived as problematic, and that the course was discontinued after a few terms. A new course was later created for supervision and the role of teaching in IPL, where, however, it was required that teachers had taken other higher education courses in order to be qualified.

It was clear from study guides and supervisor guides of the interprofessional modules, before the current curriculum was staged (i.e., phases 1–2), that there was and still are (i.e., in phase 4), high expectations for these particular teachers. An assumption that pervades the quote below, is that these supervisors was expected to possess a high level of competence in terms of the pedagogical model of PBL, the content, and the supervision of different groups, and were also expected to take responsibility for actively further developing these skills:

*‟Most of you are very experienced as supervisors … Those of you who are new, take help from your experienced colleagues and feel free to contact us if you have questions … It is important that you − are knowledgeable in problem-based learning, feel confident in your role as supervisor and have solid experience of being a supervisor in problem-based learning groups … have knowledge and interest in the content and take responsibility for further developing this knowledge, for example by attending the lectures.”* [Study guide with supervisor comments, the first IPL module, spring semester 2016, phase 2.]

Also, other documents stated that the teachers who work within the interprofessional modules should have good competence in both PBL and IPL. The introduction of a mentoring system where experienced teachers support new teachers, was highlighted as a recommended strategy in an early report (phase 1), but does not seem to have had an impact as is does not seem to have been implemented. Several letters of comment from phase 1 pointed towards the need for resources for competence development regarding the supervision of IPL. But beyond support structures such as supervisor meetings and study guides for teachers, such support seems sparse.

## Discussion

This study aimed to study intentions and outcomes of a revised interprofessional education curriculum, developed to educate HPE students in interprofessional competencies, using a theory-based evaluation. In particular, we were interested in why and how the curriculum was revised (research question 1), how the intentions and outcomes could be theorized in Lees model [14] for curriculum development (research question 2), and what lessons were to be learnt from this work (research question 3). Using theory to analyse the data was important to make sure to recognize the multi-dimensional and dynamic context of several different stakeholders that surrounds the curriculum [14].

### A curriculum in pace with healthcare needs

The findings of this study identify a variety of aspects relating to the *why* and *how* of curriculum revision (research question 1), such as what drives a change like this and what facilitates making it work. The importance of IPL is visible throughout the documents; this is in line with contemporary research which recognizes the need to connect curriculums to larger political, economic, and social issues surrounding the context whereby the students are about to work in their future professions [14]. A systematic review demonstrated a positive impact of IPL in healthcare systems, in terms of improving HPE program students’ knowledge, skills, and attitudes towards collaborative teamwork [22]. One of the major arguments for the revision of the curriculum in this study was to ensure high quality IPL by keeping pace with the demands of current healthcare, because IPL is considered essential for present and future healthcare professionals and their patients, relating to Lees’ [14] first dimension regarding future orientation but also affecting the third on learning activities. The latter is identified in the results of this study, as the planning of the IPL modules was impacted by factors outside of the university, for example the healthcare sector, which forms a strong voice as stakeholder in how the educational content is enacted in the healthcare context. For example, the initial suggestion from the university to change the content in the second module from improvement science to stroke simulation was criticized by the healthcare sector, and later, the suggested changes were withdrawn from the curriculum. The second and third modules in the IPL curriculum include cooperation between students, faculty, and healthcare, which is clearly a valuable stakeholder with regard to making the curriculum work. This might explain why some of the outcomes for the curriculum revision did not turn out the way that initially was intended, relating to the second research question. Historical and cultural forces influence the kind of reshaping of current curriculum that it is possible to implement [14], and it is crucial to acknowledge these factors and consider how to address these issues. In this case, the university seemed to observe the objections and decided not to go through with the suggested changes.

### Organizational facilitators for increasing legitimacy of curriculum development

The findings also identify facilitating organizational factors that we can learn from (research question 3), essential for providing legitimacy and cooperation during the revision. For instance, the introduction of a programme director for IPL, and the occurrence of some committed leaders on the respective HPE programs. Similar findings are described in other studies where a coordinator for interprofessional activities was considered important for progress and implementation, and that the support from the management of the departments and faculties highlighted IPL as equally important as other pedagogical activities [23–26]. In this study’s setting, however, IPL has been a natural feature for decades, and legitimacy has also, to some extent, existed before, although current legitimacy may have been further rooted along with the revision of the curriculum. Loughlin et al. [26] highlight a similar function as described above but refer to “change champions”, i.e., people in senior position leading the change and promoting the cause [26].

Friction and resistance during curriculum revision may cause power struggles and difficulties in cooperation, not least between departments and HPE programs. In this study we discovered that IPL modules today are more coherently described in course documents. This is potentially a consequence of the coordination and leadership within IPL, and thus primarily relates to Lees’ [14] fourth organization-focused dimension but also influences the structure of learning outcomes, i.e., dimension two. There are indeed challenges related to IPL curriculum development as these tend to span over multiple departments and/or faculties, as well as geographical locations, requiring solid arguments and mutual goals to bridge these silo-like structures [23, 27]. IPL has been described as a parallel topic that tends to be less prioritized than profession focused content [28]. Current literature on IPL commonly focuses on students learning outcomes and factors that may facilitate, or hinder, implementation [6, 7] rather than *how* a curriculum is created, revised or performed. This study, thus, contributes to the field by providing an example of how a curriculum revision in IPL can be carried out and how it may drift over time.

### The balance between pedagogical innovation and pragmatism, and the requirements of teachers

As in all curriculum development, independent of subject and content, there is an important element that regards the involved teachers who are expected to carry out the desired changes. The pedagogical models used as well as resources and competence of the team of teachers, are essential for how a curriculum revision is accepted and performed. Research findings emphasizes that the time available, the understanding of the change, and the level of commitment of the teachers involved in the change [11, 26], as well as using realistic cases [29], are crucial for creating high quality curriculum. In this study, the pedagogical finesse with, for instance, the “cut-outs” was to create learning situations for the students that would develop their interprofessional competence and integrate the professional content with the interprofessional content of a course. Such integration has been pointed at as being inextricably intertwined in IPL curriculum development, requiring a high degree of awareness [11]. As this learning activity was considered too complicated by teachers and removed, we can conclude, in accordance with statements from the interview data, that there is a fine line between pedagogical creativeness and pragmatism, that can be related to both the fourth and third dimension of Lees’ [14] model; i.e., what is beneficial from a learning perspective for students may not be practically feasible for the team of teachers, at least not immediately (research questions 2–3). Creative pedagogical models for teaching students the content of IPL may need time to settle, and resources for proper implementation. This study can also conclude that the faculty had high expectations on the competences of the teachers who were supposed to work according to the renewed curriculum, but there were few activities described in the documents that actively supported the teachers to reach those high expectations. It is argued in the literature that the success of a revised curriculum rests heavily on the teachers who are the ones who should put the reforms into practice [30]. It is important to educate teachers how to use new educational tools and how to properly introduce them to the students. Also, as some of the teaching staff in Swedish HPE are clinical practitioners, finding time to take part in pedagogical developments may prove challenging. There is a need for future research to further explore how we can facilitate participation in the IPL modules for clinical practitioners, so that these teachers have appropriate resources to let students benefit from their competence and experience.

More research is needed regarding the teachers’ and students’ perspectives on the curriculum revision in this study, in order to gain an understanding of the extent to which teachers were “on board” when the changes were implemented, and what improvement suggestions they might have on the performance of this curriculum revision (research question 3).

#### Methodological considerations

This was a document study which provided a unique insight into other components of the lived experience [17]; in this case, intentions and outcomes of the development of a revised curriculum in IPL. As in all qualitative research, considerations must be made regarding how to evaluate spoken or unspoken responses [17]. A written word may have different meanings in different contexts, which naturally increases the possibility of different interpretations compared to the spoken word [17]. In a qualitative document study, the documents’ relevance must be evaluated as well as their representativeness and authenticity [18], along with evaluating the study’s trustworthiness in terms of transferability, credibility, dependability and confirmability [16]. To begin with, the documents were considered relevant since they mirror the intentions of decision-makers as well as instructions for teachers and students, and thus, provide a multifaceted picture of the phenomena of curriculum development. The individual interviews contributed to supplementary data, to an increased understanding of the process and to further strengthening the analysis.

## Conclusions

This study has identified important lessons to be learnt when revising an existing fully-integrated IPL curriculum in health professions education programs. The main ambition for this IPL curriculum revision was to legitimize and provide education that is up to date with current healthcare needs, and which provides students with competencies to collaborate in teams to ensure patient safety. When redesigning a curriculum, there seems to be a fine balance between pedagogical innovation and pragmatism. Orchestrating a curriculum that is both a creative learning activity for students and comprehensible for teachers with a variety of experiences, is no easy task. This study identified that the links between organizational support structures provided and the expectations put on teachers were not aligned.

### Supplementary Information

**Supplementary Material 1.**

## Data Availability

The documents are available upon request from the corresponding author. The key individuals, however, have only consented to participate in studies conducted by this research group, and we thus lack their consent to share the interview data.
