# Supplementary material for: Exploring a revised interprofessional learning curriculum in undergraduate health education programs at Linköping University
Source: BMC Med Educ. 2024 Apr 26;24:466. doi: 10.1186/s12909-024-05458-3 (PMC11055219; doi:10.1186/s12909-024-05458-3)
Supplement: Supplementary file 1 — Supplementary Material 1. [file 12909_2024_5458_MOESM1_ESM.docx]

**Appendix 1. Interview guide to key persons**

1. Please describe your role during the development of the revised IPL curricula
2. What do you think were the intentions with the revision? What, according to you, were the important path choices? When, and why?
3. From your point of view, what were the challenges and success factors during the process? Lessons learned?
4. From the document analysis: we have identified some aspects in the original revised IPL curricula or the initial report “In pace with the Future” that are no longer visible in the current documents. Do you agree? If yes, what do you perceive as the reasons behind the shifts over time?

- Portfolio
- Number of seminars during the first IPL module
- “Cut-outs” scenarios
- Mentoring system and other support structures for teachers, competency requirements
- Opportunities to interprofessional learning outside the three IPL-modules
- Also, the change from stroke simulation as subject for the second IPL module (as suggested in the report) to improvement science (revised curricula)

1. One of the initial intentions were to integrate the IPL competency as a part of the professional competency. How do you think it turned out? Why?
2. The revised curricula entailed that the introduction of problem-based learning was assigned to the different programs. How do you think it turned out? Pros and cons?
